# Antimicrobial use and stewardship opportunities in Burkina Faso: findings from the first Global Point Prevalence Survey in eight hospitals

**DOI:** 10.1017/ash.2026.10294

**Published:** 2026-02-05

**Authors:** André Yi Yè Aburi Nagalo, Ann Versporten, Odilon D. Kaboré, Orokia S. Momo, Noutin F. Michodigni, Bintou Sanogo, Jacques Zoungrana, Ines Pauwels, Ibrahim Traoré, Armel Poda, Mahamoudou Sanou, Herman Goossens, Abdoul-Salam Ouedraogo

**Affiliations:** 1 Laboratoire National de Référence des résistances aux antimicrobiens, Bobo-Dioulasso, Burkina Faso; 2 Laboratoire d’analyses biomédicales, Centre Hospitalier Universitaire Régional de Ouahigouya, Ouahigouya, Burkina Faso; 3 Laboratory of Medical Microbiology, University of Antwerp, Antwerp, Belgium; 4 Instituts des sciences de la santé, Université Nazi BONI, Bobo-Dioulasso, Burkina Faso; 5 Unité de bactériologie-virologie, Centre Hospitalier Universitaire Pédiatrique Charles de Gaulle, Ouagadougou, Burkina Faso; 6 Unité de Formation et de recherche en Sciences de la Santé, Université Joseph Ki-Zerbo, Ouagadougou, Burkina Faso; 7 Département de Pédiatrie, CHU Sourô Sanou, Bobo-Dioulasso, Burkina Faso; 8 Service des maladies infectieuses, CHU Sourô Sanou, Bobo-Dioulasso, Burkina Faso; 9 Service de réanimation, CHU Sourô Sanou, Bobo-Dioulasso, Burkina Faso

## Abstract

**Objective:**

To describe the prevalence, patterns, and quality indicators of antimicrobial use (AMU) in Burkinabe hospitals and identify priorities for stewardship.

**Design:**

Multicentre, cross-sectional point prevalence survey (PPS) following the Global Point Prevalence Survey protocol.

**Setting:**

Eight public hospitals across six cities in Burkina Faso (3 primary, 3 secondary, and 2 tertiary), February–June 2019.

**Participants:**

All inpatients present on the survey day at 8:00. Medical records were reviewed for those receiving systemic antimicrobial agents.

**Methods:**

Standardized ward- and patient-level data were collected on indications, agents, routes, and WHO AWaRe categories, alongside quality indicators (documented indication, stop/review date, guideline compliance, and targeted vs empirical therapy). Descriptive analyses compared hospital tiers.

**Results:**

Of 994 inpatients, 729 (73.3%) received ≥1 antimicrobial (range by tier: tertiary 69.7%, secondary 79.2%, primary 79.2%). Community-acquired infections accounted for 96.0% of therapeutic indications. Leading reasons were skin/soft tissue infections (12.2%), gastrointestinal infections (10.7%), and pneumonia (10.4%). Parenteral administration predominated. The most used agents were ceftriaxone (27.8%), metronidazole (15.7%), and gentamicin (9.4%). By AWaRe, Access agents comprised ∼ 50%, Watch agents ∼ 50% overall, with higher Watch use in tertiary hospitals; no Reserve antibiotics were recorded. Quality indicators were suboptimal: the indication was documented in 15.6%, the stop/review date was absent in 93.0%, and 41.1% of prescriptions were guideline-compliant. Therapy was largely empirical (98.5%). Prolonged surgical prophylaxis (>1 day) represented 86.8% of prophylaxis courses.

**Conclusions:**

Antimicrobial use (AMU) prevalence in Burkinabe hospitals is high, dominated by empirical therapy and Watch-class ceftriaxone. Deficits in prescribing quality and limited diagnostic use highlight urgent needs for context-adapted antimicrobial stewardship: enforce guideline-concordant care, curb prolonged prophylaxis and unnecessary Watch-class use, and expand affordable microbiology capacity to enable targeted therapy.

## Introduction

Antimicrobial resistance (AMR) is a major global health threat that undermines decades of medical progress. It limits effective treatment options, leading to increased morbidity, mortality, and healthcare costs.^1^ In 2019, 4.95 million deaths were associated with bacterial AMR, including 1.27 million deaths directly attributable to bacterial AMR. Low- and middle-income countries (LMICs), particularly those in the Western African region, are among the most affected, with an AMR burden estimated at 27.3 deaths per 100,000 inhabitants.^2^ Excessive and inappropriate antimicrobial use (AMU) remains a primary driver of AMR. In some LMIC hospitals, up to 90% of inpatients receive antibiotics, many without clear indication or adherence to guidelines. Understanding prescribing patterns through systematic surveillance is therefore essential to guide antimicrobial stewardship (AMS) interventions.^3–5^ To address this misuse, surveillance of AMU is crucial, as it provides a better understanding of how antimicrobials are used, investigates current practices, and highlights areas for improvement.^1,6^ Point prevalence surveys (PPSs) are widely recognized surveillance methods that provide valuable information on antimicrobial prescribing practices and other relevant factors in hospitalized patients, with limited resources required.^7–10^ The Global Point Prevalence Survey (Global-PPS) of Antimicrobial Consumption and Resistance (Global-PPS, www.global-pps.com) is a standardized methodology that aims to assess the global prevalence of antimicrobial prescription, healthcare-associated infections, and resistance in hospitals and healthcare centers worldwide. It is designed to be feasible also in countries with low resources, support, and expertise. This methodology, developed by the University of Antwerp, has been used to assess AMU in more than 1,600 unique institutions across 100 countries worldwide.^11^ The first Global-PPS survey was conducted in 2015 among 53 countries and revealed that AMU varied widely between continents and countries, reaching three-quarters of inpatients treated in some African countries. In 2017, Burkina Faso, a West African nation, adopted its National AMR Action^12^ and highlighted its commitment to the global fight against AMR by ensuring effective AMR surveillance and optimizing the antimicrobial prescription in hospital settings. This study, to the best of our knowledge, represents the first effort to evaluate antimicrobial prescribing practices in selected primary, secondary, and tertiary hospitals in Burkina Faso.

## MEthodology

### Study design

A multicentre PPS on AMU was conducted using the standardized, freely available **Global-PPS** protocol and web-based data collection platform (www.global-pps.com). The survey was carried out between **February and June 2019** across participating hospitals. In each ward, data were collected on a single designated day, including all inpatients occupying a bed at **8:00 a.m.** on the day of the survey. Due to logistical and staffing constraints, hospitals conducted their surveys on different days within this period, although the standardized methodology ensured data comparability across sites. Trained **general practitioners**, acting as local investigators, were responsible for data collection and entry into the secure Global-PPS online system. Training sessions were organized to ensure consistent application of definitions and procedures across hospitals.

Data were collected on key variables, including **patient demographics (age, sex)**, **type and route of antimicrobial used**, **indication for therapy**, and **classification of infection** as either *community-acquired (CAI)* or *healthcare-associated infection (HAI)*. Prescriptions for **surgical and medical prophylaxis** were also recorded.

The WHO AWaRe (Access, Watch, Reserve) classification, developed by the World Health Organization, was used to categorize prescribed antibiotics in this study. This framework groups antibiotics according to their spectrum of activity, risk of promoting AMR, and recommended clinical use: Access antibiotics are first- or second-line agents for common infections with a lower resistance risk; Watch antibiotics have broader spectra and higher resistance potential, requiring closer monitoring; and Reserve antibiotics are last-resort treatments for suspected or confirmed multidrug-resistant infections and should be used sparingly under strict stewardship oversight.

Additionally, a predefined set of **antimicrobial quality indicators** was assessed, including documentation of the **indication** and **stop/review date**, the **availability of local prescribing guidelines**, and **compliance** with these guidelines. Built-in validation checks within the Global-PPS platform and centralized oversight ensured the accuracy and completeness of the collected data.

### Study settings

**Figure 1.**
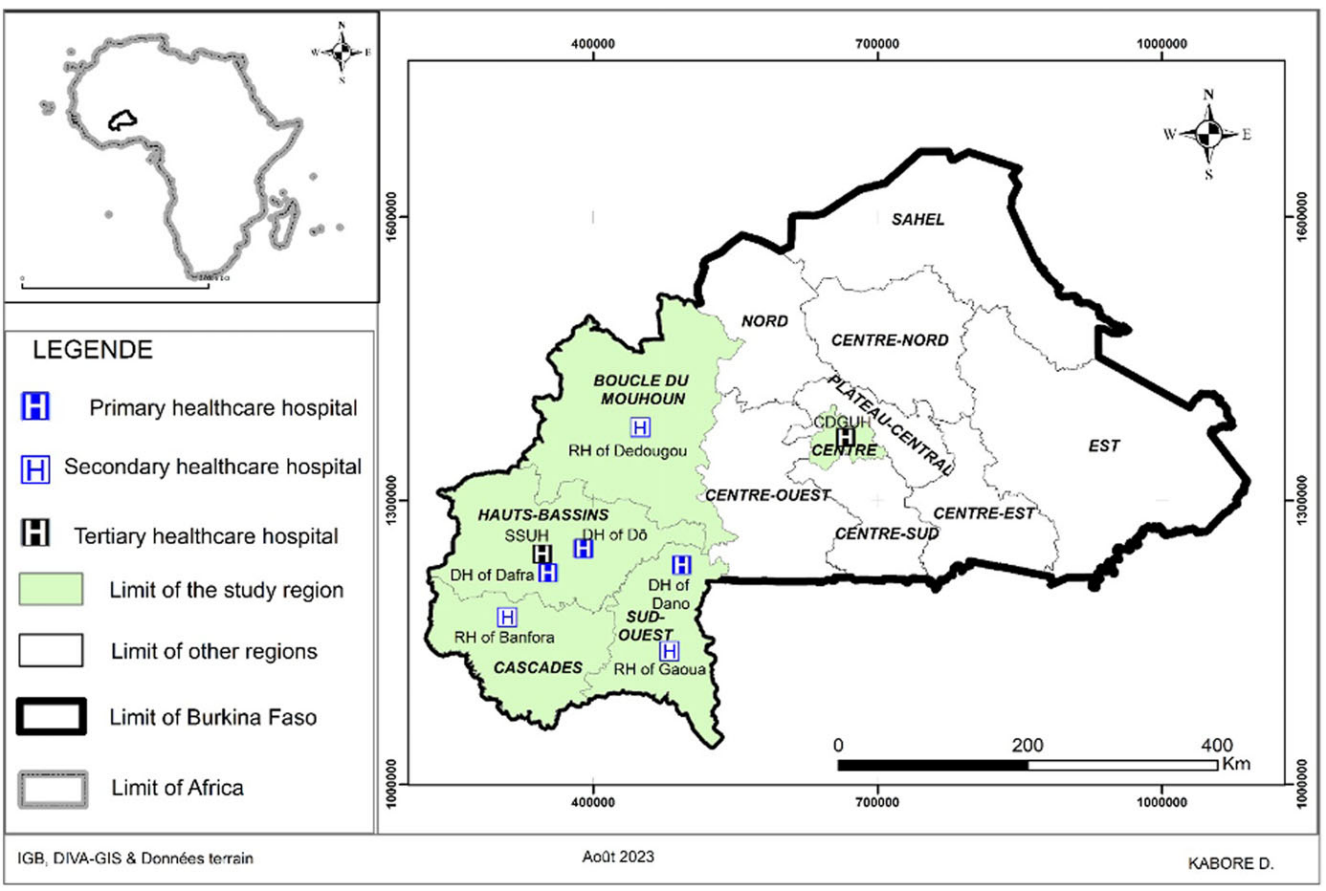
Map of Burkina Faso showing the surveyed hospitals (KABORE D., 2023). DH, district hospital; RH, regional hospital; SSUH, Sourô Sanou University Hospital; CDGUH, Charles de Gaulle University Hospital.

This first Global-PPS in Burkina Faso was conducted across eight hospitals located in six cities, including the two main urban centers, Ouagadougou and Bobo-Dioulasso. The study included the main public hospitals in the western and southern parts of the country (4 health regions) and one specialized hospital in the central region (Ouagadougou), as shown on the map (Figure 1). The healthcare structure in Burkina Faso, comprising 49 primary, nine secondary, and six tertiary hospitals, illustrates the uneven distribution of diagnostic and human resources, with primary and secondary hospitals facing the greatest constraints.

Together, the selected hospitals accounted for approximately **12% of all national hospital beds**, providing a meaningful though not exhaustive snapshot of AMU across the country. The sample included:

- **Two tertiary hospitals** (Ouagadougou and Bobo-Dioulasso), one of which was a specialized pediatric and neonatal hospital.
- **Three secondary-level hospitals** (Banfora, Dédougou, and Gaoua) serve as regional referral centers.
- **Three primary-level hospitals**, including two in Bobo-Dioulasso and one in Dano, represent the first line of hospital-based healthcare delivery.

All hospitals had facilities for **basic biochemistry and hematology testing**, while only the **secondary and tertiary hospitals** were equipped with **microbiology services**, including bacterial culture and antimicrobial susceptibility testing. However, at the time of the survey, none of the hospitals had an **active AMS program** in place.

This selection reflects the diversity of hospital infrastructure and diagnostic capacities in Burkina Faso’s public health system and allows the survey to highlight variations in AMU across healthcare levels

### Data analysis

All data were entered directly into the **Global-PPS** web-based application and anonymized before analysis. The dataset included hospital-, ward-, and patient-level information from all eight participating hospitals, with no data excluded.

Data were extracted from the Global-PPS platform and analyzed using **Microsoft Excel (version 2019)**. Categorical variables were summarized as frequencies and percentages, while continuous variables were expressed as means with standard deviations, as appropriate. The **prevalence of AMU** was defined as the number of inpatients receiving at least one antimicrobial agent divided by the total number of inpatients surveyed on the day of data collection.

Comparisons of categorical variables between hospitals and ward types were performed using the **χ^2^ test** or **Fisher’s exact test** when expected cell counts were <5. All analyses followed standard Global-PPS methodology to ensure consistency and comparability across sites.

## Results

### Characteristics of participating hospitals and eligible patients

On the day of the survey, a total of 1,344 beds and 994 inpatients were surveyed across all eight (8) hospitals, accounting for a bed occupancy of 74.0%. The patients treated with at least one antimicrobial had an average age of 18 years and a median age of 7 years.

### Prevalence of antimicrobial use

A total of 729 out of 994 patients received at least one antimicrobial during the day of the PPS (73.3%). The prevalence of AMU varied according to hospital care levels: 71.6% (range 59.4%–92.3%) in primary, 79.2% (range 69.8%–87.1%) in secondary, and 69.7% (range 65.0%–83.0%) in tertiary facilities. Despite these differences, the variation was not statistically significant (*p*-value = .461). Additionally, no statistically significant correlation was found between bed occupancy rate and prevalence of AMU (*p*-value = .424) as summarized in Tables 1 and 6.

**Table 1.** Characteristics of wards and patients (treated with at least one antimicrobial) per hospital tier level

Characteristics N (%)	Primary hospital level		Secondary hospital level		Tertiary hospital level		Total	
**Number of hospitals**	3		3		2		8	
**Numbers of beds**	171		493		680		1,344	
**Number of inpatients**	109	(63.7)	361	(73.2)	524	(77.1)	994	(74.0)
**Number of treated patients**	78	(71.6)	286	(79.2)	365	(69.7)	729	(73.3)
Patient age								
Neonate (≤1 month)	0	(.0)	42	(14.7)	68	(18.6)	110	(15.1)
Child (>1 month–≤17 years)	34	(43.6)	128	(44.8)	174	(47.7)	336	(46.1)
Adult (≥18 years)	44	(56.4)	116	(40.6)	123	(33.7)	283	(38.8)
**Patient gender**								
F	42	(53.8)	150	(52.4)	160	(43.8)	352	(48.3)
M	36	(46.2)	136	(47.6)	205	(56.2)	377	(51.7)
**Ward activity**								
Medicine	59	(75.6)	207	(72.4)	177	(48.5)	443	(60.8)
Surgery	19	(24.4)	66	(23.1)	140	(38.4)	225	(30.9)
Intensive care	0	(.0)	13	(4.5)	48	(13.1)	61	(8.4)

Table 1 provides a summary of the patients surveyed based on the tiered level of the hospital. The study included a higher number of children than adults or neonates. Tertiary hospitals had a larger proportion of neonates (18.6%) and children (47.7%) surveyed compared to primary hospitals, where adults were more commonly encountered (56.4%). The wards where patients were surveyed varied depending on the hospital-tiered level. Patients in intensive care wards (IC) constituted the smallest proportion of those surveyed, with only 4.5% and 13.1% of patients in secondary and tertiary hospitals, respectively.

### Reason for prescribing antimicrobials

Across all hospitals, the most reported reasons for prescribing antibiotics (ATC J01) were skin and soft tissue infections (12.2%) and gastrointestinal infections (10.7%). However, there were slight variations between the hospital care levels, with pneumonia or lower respiratory tract infections being the most common reason in primary care hospitals (24.4%) and medical prophylaxis for newborn risk factors being the second most common reason in tertiary hospitals (12.1%) (Table 2). Overall, therapeutic use accounted for 90.2% of antimicrobial prescriptions, with CAI infections being the main type of infection leading to AMU (95.7%). Surgical prophylaxis of >1 day (SP3) was common in all hospitals, accounting for up to 86.8% of all prescriptions for surgical prophylaxis (Table 6).

**Table 2.** Top 12 indications of antimicrobial use per hospital tier level

	Primary hosp.		Secondary hosp.		Tertiary hosp.		Total	
Indications	*n*	(%)	*n*	(%)	*n*	(%)	*n*	(%)
SST	3	(3.8)	30	(10.5)	56	(15.3)	89	(12.2)
GI	17	(21.8)	45	(15.7)	16	(4.4)	78	(10.7)
Pneu	19	(24.4)	22	(7.7)	35	(9.6)	76	(10.4)
NEO-MP	0	(0.0)	22	(7.7)	44	(12.1)	66	(9.1)
IA	3	(3.8)	12	(4.2)	35	(9.6)	50	(6.9)
Other	0	(0.0)	21	(7.3)	26	(7.1)	47	(6.4)
Proph OBGY	17	(21.8)	25	(8.7)	3	(.8)	45	(6.2)
Malaria	7	(9.0)	17	(5.9)	15	(4.1)	39	(5.3)
CNS	1	(1.3)	8	(2.8)	18	(4.9)	27	(3.7)
SEPSIS	3	(3.8)	10	(3.5)	11	(3.0)	24	(3.3)
UNK	0	(.0)	18	(6.3)	5	(1.4)	23	(3.2)
Bron	1	(1.3)	10	(3.5)	8	(2.2)	19	(2.6)

SST, skin and soft tissue; GI, gastrointestinal infections; Pneu, pneumonia or lower respiratory tract infections; NEO-MP, Drug is used as medical prophylaxis for NEWBORN risk factors; IA, intraabdominal sepsis including hepatobiliary; Proph OBGY, prophylaxis for obstetric or gynaecological surgery; CNS, infections of the central nervous system; UNK, completely unknown indication; Bron, bronchitis; Hosp, hospital.

### Commonly used antimicrobials

**Figure 2.**
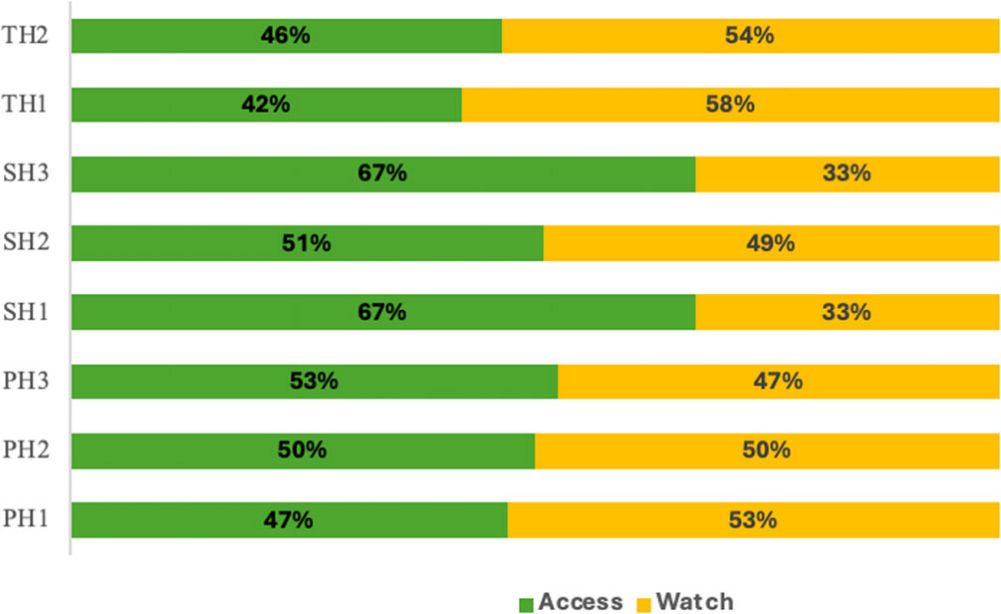
Antibiotic use according to WHO AWaRe classification. PH, primary hospital; SH, secondary hospital; TH, tertiary hospital.

Out of all antimicrobials, antibacterials for systemic use accounted for 80.6% of prescriptions. Cephalosporins (J01D) and Penicillin (J01C) were the most prescribed antibiotic classes across all the different hospital levels, comprising 35.0% and 17.7% of prescriptions, respectively (Table 3). According to the WHO AWaRE classification, antibiotics of the Access category were the most frequently prescribed and accounted for 52% of all prescriptions, while antibiotics in the Watch category were the second most frequently prescribed (48%), representing 37%, 49%, and 57% of prescriptions in secondary, primary, and tertiary hospitals, respectively. No antibiotics from the Reserve category were prescribed in any of the surveyed hospitals (Figure 2).

**Table 3.** Class of antimicrobial used according to the three healthcare levels

Antimicrobials *n* (%)	Primary hosp.		Secondary hosp.		Tertiary hosp.		Total	
**Antibacterials for systemic use (J01)**	**93**	**(70.5)**	**438**	**(80.1)**	**493**	**(83.4)**	**1,024**	**(80.6)**
Penicillins (J01C)	25	(18.9)	122	(22.3)	78	(13.2)	225	(17.7)
Cephalosporins (J01D)	46	(34.8)	155	(28.3)	244	(41.3)	445	(35.0)
Sulfonamides and trimethoprim (J01E)	1	(0.8)	3	(0.5)	6	(1.0)	10	(0.8)
Macrolides and lincosamides (J01F)	0	(0.0)	4	(0.7)	16	(2.7)	20	(1.6)
Aminoglycosides (J01G)	3	(2.3)	46	(8.4)	72	(12.2)	121	(9.5)
Quinolones (J01M)	1	(0.8)	5	(0.9)	16	(2.7)	22	(1.7)
Other antibacterials (J01X)	17	(12.9)	103	(18.8)	61	(10.3)	181	(14.3)
**Antimycotics for systemic use (J02)**	3	(2.3)	2	(0.4)	6	(1.0)	11	(0.9)
**Antimycobacterials for systemic use (J04)**	0	(0.0)	4	(0.7)	32	(5.4)	36	(2.8)
**Antivirals for systemic use (J05)**	2	(1.5)	0	(0.0)	12	(2.0)	14	(1.1)
**Antiprotozoals (P01)**	29	(22.0)	79	(14.4)	42	(7.1)	150	(11.8)
**Antidiarrheals (A07)**	5	(3.8)	24	(4.4)	6	(1.0)	35	(2.8)

Hosp, hospital.

### Common antibiotics according to the department, the age, and the indications

The top three most prescribed antibiotics were ceftriaxone (27.8%), metronidazole (15.7%), and gentamicin (9.4%) as presented in the following table (Table 4). Ceftriaxone was found to be the most frequently used antibiotic for therapy and prophylaxis, with a proportion ranging from 27.2% to 36.7% of prescriptions, averaging 24.2% in medical departments and 37.4% in surgical departments (Table 5). In addition to the top three antibiotics, a relatively high usage of ampicillin (7.3%) and amoxicillin/clavulanic acid (6.9%) was observed. Cefotaxime was commonly used in neonate patients (33.0%) and the ICU (21.4%).

**Table 4.** Top 10 antimicrobials per tiered level

Antimicrobials		Primary hosp.		Secondary hosp.		Tertiary hosp.		Total	
ATC Code	INN	n	(%)	n	(%)	n	(%)	n	(%)
J01DD04	Ceftriaxone	43	(32.6)	137	(25.0)	173	(29.3)	353	(27.8)
J01XD01	Metronidazole	18	(13.6)	114	(20.8)	68	(11.5)	200	(15.7)
J01GB03	Gentamicin	3	(2.3)	46	(8.4)	71	(12.0)	120	(9.4)
P01BE03	Artesunate	18	(13.6)	54	(9.9)	34	(5.8)	106	(8.3)
J01CA01	Ampicillin	12	(9.1)	74	(13.5)	7	(1.2)	93	(7.3)
J01CR02	Amoxicillin and EI	5	(3.8)	23	(4.2)	59	(10.0)	87	(6.9)
J01DD01	Cefotaxime	1	(0.8)	13	(2.4)	60	(10.2)	74	(5.8)
A07AA02	Nystatin	5	(3.8)	24	(4.4)	6	(1.0)	35	(2.8)
J01CA04	Amoxicillin	8	(6.1)	22	(4.0)	2	(0.3)	32	(2.5)
J01DD08	Cefixime	2	(1.5)	4	(0.7)	11	(1.9)	17	(1.3)

ATC, anatomical therapeutic chemical; EI, enzyme inhibitor; Hosp, hospital; INN, International Nonproprietary Names.

**Table 5.** Top 5 antibiotics according to department, age, and indications

	Ranks				
Variables n (%)	1^rst^	2^nd^	3^rd^	4^th^	5^th^
**Department**					
Medical	Ceftriaxone	Metronidazole	Gentamicin	Ampicillin	Cefotaxime
	200 (24.2)	104 (12.6)	78 (9.4)	64 (7.7)	52 (6.3)
Surgical	Ceftriaxone	Metronidazole	Amoxicillin+ EI	Ampicillin	Gentamicin
	127 (37.4)	84 (24.7)	39 (11.5)	27 (7.9)	24 (7.1)
Intensive care	Ceftriaxone	Cefotaxime	Gentamicin	Metronidazole	Amoxicillin+ EI
	26 (25.2)	22 (21.4)	18 (17.5)	12 (11.7)	9 (8.7)
**Age groups**					
Neonates	Cefotaxime	Gentamicin	Ceftriaxone	Ampicillin	Metronidazole
	62 (33.0)	39 (20.7)	26 (13.8)	24 (12.8)	21 (11.2)
Children	Ceftriaxone	Metronidazole	Gentamicin	Ampicillin	Amoxicillin+ EI
	195 (31.2)	77 (12.3)	65 (10.4)	39 (6.2)	21 (3.4)
Adults	Ceftriaxone	Metronidazole	Amoxicillin+ EI	Ampicillin	Gentamicin
	132 (28.9)	102 (22.3)	64 (14.0)	30 (6.6)	16 (3.5)
**Indications**					
CAI	Ceftriaxone	Metronidazole	Gentamicin	Amoxicillin+ EI	Ampicillin
	295 (27.2)	170 (15.7)	114 (10.5)	74 (6.8)	65 (6.0)
HAI	Ceftriaxone	Amoxicillin+ EI	Metronidazole	Ciprofloxacin	Gentamicin
	17 (36.7)	9 (18.4)	7 (14.3)	4 (8.2)	3 (6.1)
Med. Prophylaxis	Ceftriaxone	Ampicillin	Amoxicillin	Amoxicillin+ EI	Metronidazole
	10 (28.6)	3 (8.6)	3 (8.6)	2 (5.7)	2 (5.7)
Surg. Prophylaxis	Ceftriaxone	Ampicillin	Metronidazole	Amoxicillin	Gentamicin
	23 (33.8)	19 (27.9)	14 (20.6)	8 (11.8)	1 (1.5)
Unknown/others	Ceftriaxone	Metronidazole	Ampicillin	Amoxicillin+ EI	Cefotaxime
	7 (21.9)	7 (21.9)	6 (18.8)	1 (3.1)	1 (3.1)

EI, enzyme inhibitor; CAI, community-acquired infection; HAI, healthcare-associated infections; Med. Prophylaxis, medical prophylaxis; Surg. Prophylaxis, surgical prophylaxis.

### Quality indicators for prescribing

During the survey, quality indicators were recorded for all prescriptions using specific criteria, as shown in the table below (Table 6). A stop/review date was not documented in the medical records for 93.0% of the antimicrobial prescriptions. The indications were not documented in 15.5% of the prescriptions. Guideline compliance for prescriptions was observed in 40.2% of cases, and varied widely among the eight hospitals, ranging from 3.0% in one tertiary hospital to 78.7% in one secondary hospital. Empirical therapy accounted for 98.3% of all prescriptions.

**Table 6.** Overall antimicrobial use prevalence N = 1,270

Characteristics N (%)	PH1		PH2		PH3		SH1		SH2	SH3			TH1		TH2		
Levels of healthcare	Primary		Primary		Primary		Secondary		Secondary	Secondary			Tertiary		Tertiary	Total	
**Total beds**	47		71		53		188		130		175		526		154	**1,344**	
**Hospitalized patients**	32	68.1%	51	71.8%	26 49.1%		159	84.6%	86	66.2%	116	66.3%	389	74.0%	135 87.7%	**994**	**74.0%**
**Treated patients**	19	59.4%	35	68.6%	37 142.3%		111	69.8%	74	86.0%	101	87.1%	253	65.0%	98 72.6%	**728**	**73.2%**
**Prescribed antiotics**	28		48		79		216		136		195		401		167	**1,270**	
**Number of prescribed antibiotics/patients**	1.47		1.37		2.14		1.95		1.84		1.93		1.58		1.70	**1.74**	
**Ward activity** medical	16	84.2%	22	62.9%	34	91.9%	98	88.3%	37	50.0%	72	71.3%	138	54.5%	46 46.9%	**463**	**63.6%**
Surgical	3	15.8%	13	37.1%	3	8.1%	13	11.7%	27	36.5%	26	25.7%	95	37.5%	47 48.0%	**227**	**31.2%**
Intensive care	0	.0%	0	.0%	0	.0%	0	.0%	10	13.5%	3	3.0%	20	7.9%	5 5.1%	**38**	**5.2%**
**Patient gender** female	7	36.8%	23	65.7%	16	43.2%	66	59.5%	36	48.6%	48	47.5%	116	45.8%	40 40.8%	**352**	**48.4%**
Male	12	63.2%	12	34.3%	21	56.8%	45	40.5%	38	51.4%	53	52.5%	137	54.2%	58 59.2%	**376**	**51.6%**
**administration** oral	10	35.7%	5	10.4%	17	21.5%	41	19.0%	29	21.3%	29	14.9%	100	24.9%	26 15.6%	**257**	**20.2%**
Parenteral	18	64.3%	43	89.6%	62	78.5%	175	81.0%	107	78.7%	166	85.1%	301	75.1%	141 84.4%	**1,013**	**79.8%**
**Indication** therapeutic use	23	82.1%	37	77.1%	73	92.4%	189	87.5%	121	89.0%	164	84.1%	382	95.3%	157 94.0%	**1,146**	**90.2%**
Prophylaxic use	5	17.9%	11	22.9%	6	7.6%	20	9.3%	10	7.4%	25	12.8%	15	3.7%	10 6.0%	**102**	**8.0%**
Unknown		.0%	7	14.6%		.0%	5	2.3%		.0%	6	3.1%	4	1.0%	.0%	**22**	**1.7%**
**Prophylaxis** medical	3	60.0%	1	9.1%		.0%	16	80.0%		.0%	3	12.0%	7	46.7%	4 40.0%	**34**	**33.3%**
Surgical	2	40.0%	10	90.9%	6 100.0%		4	20.0%	10 100.0%		22	88.0%	8	53.3%	6 60.0%	**68**	**66.7%**
**Surgical prophylaxis** single dose		.0%		.0%	1 16.7%			.0%	.0%			.0%		.0%	1 16.7%	**2**	**2.9%**
One day		.0%		.0%	1 16.7%		3	75.0%	2 20.0%			.0%	1	12.5%	.0%	**7**	**10.3%**
More than one day	2 100.0%		10 100.0%		4 66.7%		1	25.0%	8 80.0%		22 100.0%		7	87.5%	5 83.3%	**59**	**86.8%**
**Infection**community-acquired	22 100.0%		36 97.3%		73 100.0%		189 100.0%		110 93.2%		152 95.6%		362	94.8%	148 94.3%	**1,092**	**96.0%**
Hospital-acquired	0.0%		1 2.7%		0.0%		0.0%		8 6.8%		7 4.4%		20	5.2%	9 5.7%	**45**	**4.0%**
**Reason in notes** yes	9 32.1%		5 10.4%		2 2.5%		62 28.7%		29 21.3%		22 11.3%		55	13.7%	14 8.4%	**198**	**15.6%**
No	19 67.9%		43 89.6%		77 97.5%		154 71.3%		107 78.7%		173 88.7%		346	86.3%	153 91.6%	**1,072**	**84.4%**
Empirical therapy	28 100.0%		48 100.0%		79 100.0%		216 100.0%		136 100.0%		194 99.5%		391	97.5%	159 95.2%	**1,251**	**98.5%**
Targeted therapy	0.0%		0.0%		0.0%		0.0%		.0%		1 .5%		10	2.5%	8 4.8%	**19**	**1.5%**
**Compliance** Yes	15 53.6%		8 16.7%		17 21.5%		131 60.6%		109 80.1%		93 47.7%		144	35.9%	5 3.0%	**522**	**41.1%**
No	13 46.4%		40 83.3%		62 78.5%		85 39.4%		27 19.9%		102 52.3%		257	64.1%	162 97.0%	**748**	**58.9%**

PH, primary hospital; SH, secondary hospital; TH, tertiary hospital.

## Discussion

Antimicrobial resistance (AMR) remains a pressing global challenge, exacerbated by the overuse and misuse of antibiotics in both inpatient and outpatient settings. Low- and middle-income countries (LMICs) face disproportionate challenges due to limited resources, inadequate diagnostic capacities, and unregulated access to antibiotics.^2,6,13,14^

This first national Global-PPS in Burkina Faso revealed an alarmingly high prevalence of AMU, with 73.3% of hospitalized patients receiving at least one antimicrobial (69.7%–79.2% across facilities). These rates are more than double the global average (34.4%) and markedly higher than those in high-income countries (23.7%–62%)^3^ and neighboring Ghana (55%),^9^ though similar to Nigeria (70.7%)^5^ and parts of Asia (up to 85.7%).^3^ These high AMU rates reflect systemic pressures, i.e, empirical treatment due to limited diagnostics, insufficiently trained personnel, and weak stewardship oversight, particularly in primary and secondary hospitals. These findings underscore the urgent need to strengthen hospital governance, prescriber education, and laboratory support to promote rational antibiotic use.^13,15,16^

Most antibiotics (95.7%) targeted CAI infections, predominantly skin/soft-tissue (12.2%), gastrointestinal (10.7%), and pneumonia (10.4%). These findings mirror trends in Egypt^17^ and other LMICs, where CAIs dominate due to the burden of infectious diseases linked to poor sanitation and hygiene.^5,13^ Parenteral administration was predominant, reflecting clinical habits common in resource-limited settings.^9^

**Ceftriaxone, metronidazole,** and **gentamicin** were the most frequently prescribed antibiotics, consistent with regional studies.^18,19^ Beta-lactams were the dominant class, as reported elsewhere,^3,5,9,20,21^ though over-reliance on these drugs raises concern, given the rising resistance to third-generation cephalosporins. The heavy reliance on Watch-group antibiotics, such as ceftriaxone, despite national guidance restricting its use in primary care, reveals gaps in prescriber adherence and enforcement of stewardship policies. Encouragingly, Reserve-group antibiotics were rarely used, likely due to their high cost and limited availability in the absence of national health insurance. Strengthening regulatory enforcement and promoting adherence to national guidelines remain crucial to preserve antibiotic efficacy.

Quality indicators further revealed suboptimal prescribing practices: nearly all prescriptions lacked review or stop dates, and 15% lacked documented indications. Guideline compliance averaged only 40%, varying by hospital level. Interestingly, adherence tended to be higher in lower-level facilities. Variations across hospital levels may reflect differences in clinician experience and case complexity. While general practitioners in primary and secondary hospitals often rely more strictly on standard protocols, tertiary clinicians managing complex cases may deviate due to the lack of specialized local guidance.

A key barrier identified was the limited use of microbiological diagnostics, with 98.5% of prescriptions empirically based. Despite laboratory capacity in tertiary and some secondary hospitals, utilization remained low due to test costs, lack of insurance coverage, and frequent pretreatment with antibiotics that compromise culture results. To address this, the National AMR Action Plan (2024–2027) prioritizes upgrading laboratories, piloting microbiology units in primary care, and expanding the national AMR surveillance network from 15 to 26 sentinel sites.^22^ These actions aim to enhance diagnostic-guided therapy and strengthen evidence-based stewardship.^13,15,16^

Following this initial survey, several national AMS initiatives have been rolled out. These include the National Antibiotic Prescription Guideline (first released in 2021, revised in 2023), integrating WHO AWaRe principles, and the National Action Plan for Rational Antimicrobial Use in Hospitals.^12,23^ These guidelines have been tailored to local epidemiology and informed by data from the national laboratory-based AMR surveillance network.^24^ Prescribers from secondary and tertiary hospitals have received training on applying the national guidelines and integrating the AWaRe classification into their decision-making processes. Additionally, since 2017, an average of 500 healthcare professionals across Francophone West and Central Africa have been trained during an Inter-University Diploma course on AMR (www.diu-antibio.org/), organized annually by national universities and partners. Approximately half of these participants have come from all healthcare levels of the Burkinabe healthcare system, ensuring broad capacity building for evidence-based prescribing decisions. In parallel, Pharmaceutical and Therapeutic Committees have been institutionalized in all tertiary and secondary hospitals, some extending to primary care, to oversee AMS implementation and individualized drug dispensation. The national pharmaceutical regulatory authority now conducts annual antimicrobial consumption surveys to monitor progress. A second round of the Global-PPS is ongoing and will evaluate progress and guide corrective actions.

Implementation of the first PPS faced expected challenges. Initial apprehension among clinicians, concerned that results might expose poor practices, was mitigated by **Ministry of Health** endorsement and strong engagement of hospital managers. Mobilizing young general practitioners as data collectors and using the standardized **Global-PPS online platform** facilitated efficient data collection and validation. However, funding constraints limited geographic coverage and individualized feedback. Key findings were instead disseminated through **quarterly national AMS meetings**, ensuring that results informed national interventions.

Despite limitations, ie., non-random sampling, regional concentration, and inclusion of only ∼ 12% of national hospital beds, this survey provides baseline data for **Burkina Faso’s AMS strategy**. It has directly influenced policy design, stewardship training, and laboratory strengthening. Repeat PPS rounds, currently underway, will assess progress in stewardship implementation and identify persistent barriers to rational antibiotic use.

Overall, this first nationwide survey revealed extensive AMU in Burkinabe hospitals, largely empirical and driven by system-level constraints. Nonetheless, it has catalyzed policy reforms, guideline development, and stewardship actions that lay a foundation for sustained improvements in antimicrobial rationalization. Repeating the PPS will be essential to measure progress and ensure continued momentum toward responsible antibiotic use essential for long-term containment of AMR in Burkina Faso.

## Data Availability

Data supporting the findings of this study are available in the Global-PPS repository and can be accessed upon reasonable request to the coordination team at the University of Antwerp.
